# Association of the Extent of Internet Use by Patients With Cancer With Social Support Among Patients and Change in Patient-Reported Treatment Outcomes During Inpatient Rehabilitation: Cross-sectional and Longitudinal Study

**DOI:** 10.2196/39246

**Published:** 2023-05-17

**Authors:** Lukas Lange-Drenth, Holger Schulz, Gero Endsin, Christiane Bleich

**Affiliations:** 1 Department of Medical Psychology University Medical Center Hamburg-Eppendorf Hamburg Germany; 2 Diana Klinik Bad Bevensen Germany

**Keywords:** internet, internet use, social support, perceived social support, inpatients, patient-reported outcome measures, cancer, rehabilitation, distress, fatigue, pain

## Abstract

**Background:**

Given the increasing number of cancer survivors and their rising survival rates, rehabilitation plays an increasingly important role. Social support among patients is an essential element of inpatient and day care rehabilitation. The internet can empower patients with cancer to become more active health care consumers and facilitate information and supportive care needs. By contrast, therapists suspect that high internet use during rehabilitation may severely limit social interactions between patients, thus interfering with the patients’ rehabilitation program and jeopardizing treatment success.

**Objective:**

We hypothesized that the extent of internet use would be negatively related to social support among patients with cancer during their clinical stay as well as fewer improvements in patient-reported treatment outcomes from the first to the last day of their clinical stay.

**Methods:**

Patients with cancer participated during their inpatient rehabilitation. Cross-sectional data, such as the extent of participants’ internet use and perceived social support among patients, were collected during the last week of their clinic stay. The treatment outcomes, that is, participants’ levels of distress, fatigue, and pain, were collected on the first and last day of the clinic stay. We used multiple linear regression analysis to study the association between the extent of internet use and social support among patients with cancer. We used linear mixed model analyses to study the association between the extent of internet use by patients with cancer and the change in patient-reported treatment outcomes.

**Results:**

Of the 323 participants, 279 (86.4%) participants reported that they used the internet. The extent of the internet use (*t*_315_=0.78; *P*=.43) was not significantly associated with the perceived social support among the participants during their clinical stay. In addition, the extent of participants’ internet use during their clinical stay was not associated with changes in participants’ levels of distress (*F*_1,299_=0.12; *P*=.73), fatigue (*F*_1,299_=0.19; *P*=.67), and pain (*F*_1,303_=0.92; *P*=.34) from the first to the last day of their clinical stay.

**Conclusions:**

The extent of internet use does not seem to be negatively associated with the perceived social support among patients with cancer or with the change in patients’ levels of distress, fatigue, or pain from the first to the last day of their clinical stay.

## Introduction

### Background

Cancer survivors can experience long-term physical and psychological consequences of cancer and its treatment [1-3]. Fatigue, pain, and distress are among the most frequently reported symptoms during and after primary cancer treatment [4-9]. Given the increasing number of cancer survivors and rising survival rates resulting from progress in early detection, treatment, and cancer management [10,11], rehabilitation is playing an increasingly important role.

Different rehabilitation approaches are being used for patients with cancer worldwide. On the basis of the biopsychosocial model of the World Health Organization, these programs are based on a similar multidisciplinary understanding of cancer rehabilitation [12-14]. In Germany, after primary treatment, every patient with cancer is legally entitled to participate in a 3-week combined multidisciplinary treatment program consisting of physical therapy, patient education, relaxation training, functional training, psycho-oncological treatment, nutrition counseling, and occupational counseling, depending on the patient’s functioning and needs as assessed at the beginning of the rehabilitation [12,15]. A special feature is that in Germany, cancer rehabilitation is mainly performed in inpatient clinics [12]. Uncontrolled before-and-after studies showed that patients undergoing cancer rehabilitation can improve their somatic status, psychosocial status, and quality of life and reduce their anxiety, depression, and distress from the beginning to the end of inpatient rehabilitation [16-18].

The 2 essential elements of inpatient and day care rehabilitation are social support from other patients in cancer rehabilitation and physical activity [12]. Social support has been recognized as an important factor in overall well-being [19,20] and has been positively associated with both improvement in cancer-related stress [21] and posttraumatic growth in patients with cancer [22]. In inpatient and day care, patients in cancer rehabilitation receive social support from other patients undergoing rehabilitation with a cancer diagnosis (peer support) during therapist-guided group treatment sessions and unguided peer support during leisure-time activities. The three main attributes of peer support are (1) emotional support by discussing personal difficulties, (2) informational support by providing knowledge relevant to problem-solving, and (3) appraisal support such as encouragement to persist in problem-solving and reassurance that efforts will lead to positive outcomes [23]. Previous research found gender and age differences in seeking and providing social support. Women seem to provide more emotional support to both men and women, and they seem to receive more help in return [24]. Older people (aged ≥60) are less likely to explicitly ask for emotional support compared with younger people [25]. Systematic reviews that explored the benefits of one-on-one and group peer support interventions for patients with cancer, conducted analog and on the web, showed mixed results. Peer support interventions increased perceived distress, quality of life, and treatment-related compliance of patients with breast cancer [26], as well as the emotional health, quality of life, coping and psychosocial functioning [27,28], and empowerment of patients with cancer [29]. However, unmoderated and unstructured group peer support interventions conducted on the web without peer training had no effect or even adverse effects on quality of life, distress, and depression [26,30]. In the absence of moderation or group structure, expressions of anger and fear increased, as did discussions about death and dying [30,31]. Furthermore, initial cross-sectional studies indicated that high informational support may be associated with lower cancer-related fatigue [32].

eHealth applications and the internet can empower patients with cancer to become more active health care consumers and facilitate information and supportive care needs [33-36]. First, patients with cancer can search the internet for health- or cancer-related information or solicit medical advice from their physicians via email. Intensive searches revealed that there are no publications on the prevalence of cancer-related internet searches during inpatient or day care rehabilitation. However, the prevalence of patients with cancer in a Dutch sample, 2 American samples, and a Swedish sample who used the internet ranged from 60.2% to 79.8% [34,37-39]. In advanced economies, 87% of the population uses the internet at least occasionally [40]. The internet can help patients with cancer fulfill their needs for information regarding their diagnosis, prognosis, or treatment options [37,41,42]. Patients with cancer who search the internet for cancer-related information are younger and more highly educated than those who do not search the internet [37-39]. Second, patients with cancer can use web-based communication and web-based communities for social support. Patients with cancer can access the internet anytime and from almost anywhere [43], anonymously if desired, and even patients with rare cancer types can find other patients with the same cancer type to share experiences [44]. Web-based peer support programs used in a study setting can have a positive influence on the psychosocial well-being of patients with cancer, including quality of life and distress [26,45]. Third, eHealth programs are used as independent treatment measures or to improve or assist health care services in various phases of cancer treatment [46-49]. eHealth cancer rehabilitation and aftercare programs address logistically challenged populations and commonly use elements such as education, self-monitoring, self-management training, personalized exercise programs, communication with health care providers, and communication with fellow patients [48,49].

The starting point of this study was the observations by health care professionals of the cooperating oncological rehabilitation clinic that a high level of internet use between and after rehabilitation sessions reduced social interactions between patients during their clinic stay and high levels of internet use interfered with the patients’ rehabilitation program. This observation was somewhat related to the social displacement hypothesis. The social displacement hypothesis suggests that despite increased communication opportunities, internet use is largely a nonsocial activity that competes with face-to-face interaction and is, therefore, associated with lower social involvement and psychological well-being, as indicated by the initial results from longitudinal studies [50,51]. However, the results of subsequent studies have contradicted these claims [52-54], and a meta-analysis found only a small cross-sectional association between internet use and well-being [55]. Displacement theory has also been studied more recently with social media use instead of general internet use. The results of a study using a national probability sample could not support the social displacement hypothesis for social media use [56]. Instead, study results suggest that social media use displaces time spent using other media [57]. Results of a meta-analysis of cross-sectional studies [58] and results of longitudinal studies [59,60] indicate that the association between internet or social media use and well-being varies by the type of internet and social media use. Positive associations were found for media use directed at a specific person through which emotional information can be conveyed, such as phone calls or texting with emojis [58-60]. Furthermore, while the use of social media in general had a small negative association with well-being, interactive aspects of social media use were positively correlated with well-being [58-60]. The associations found might also be linked to the individuals’ personality or social skills. For extraverts, internet use seems to be associated with an increase in social engagement and self-esteem and a decrease in loneliness [52]. Individuals with high levels of neuroticism who use the internet frequently to seek information seem to perceive lower levels of support [53]. However, the causal direction of these associations remains unclear [53,57].

### Objective

Health care professionals at the cooperating oncological rehabilitation clinic observed that a high level of internet use between and after rehabilitation sessions reduced social interactions between patients during their clinic stay and high levels of internet use interfered with the patients’ rehabilitation program. These observations are inconsistent with previous study results on the associations between the extent of internet use, social support, and changes in well-being. However, compared with participants of previous studies on the associations between internet or social media use, social support, and well-being, patients with cancer in inpatient rehabilitation are in a different setting. During their 3 weeks of inpatient treatment, they have no or limited face-to-face contact with their friends and family, as rehabilitation clinics are often located in rural areas distant from the patients’ homes, making personal visits difficult. Although previous research has suggested that internet use does not affect social interactions, primarily with friends and family [52,54], we believe that it might be possible for internet use to affect social interactions with relative strangers in the rehabilitation setting. In addition, the psychological and mental health of patients with cancer at the beginning of rehabilitation is significantly worse than that of the general population [17], which makes comparison difficult.

We formulated the following explorative research questions: (1) is the extent of internet use negatively associated with the perceived social support among patients with cancer during their clinical stay? (2) is the extent of internet use by patients with cancer during their clinical stay negatively associated with changes in distress, fatigue, and pain scores from the beginning to the end of inpatient cancer rehabilitation, with distress being the primary outcome?

In addition, we aimed to describe the extent and purpose of internet use by patients with cancer during their clinical stay and at home.

## Methods

### Study Design

In the cross-sectional part of the study, we obtained data using a paper-pen questionnaire to gain insight into the extent and purpose of rehabilitant internet use, their preferences for future use of eHealth or web-based programs, their perceived social support from other patients, and their physical activity during the clinic stay. For the longitudinal part of the study, medical data and 3 patient-reported outcome measures (PROMs) were collected on the first day and the last day of the clinic stay.

This study followed the recommendations of the Strengthening the Reporting of Observational Studies in Epidemiology (STROBE) statement. The STROBE statement contains 18 items that are common to cohort, cross-sectional, and case-control studies. Four checklist items (items 6, 12, 14, and 15) have specific variations according to the study design [61] (Multimedia Appendix 1).

The protocol for this study is freely available at the Open Science Framework [62] and was published before the recruitment of the first participant.

### Setting, Recruitment, and Participants

The participants were recruited during the third week of their 3-week inpatient cancer rehabilitation stay at a German rehabilitation clinic. Potential participants were approached during the patient consultation. Patients in rehabilitation were recruited between September, 2018, and February, 2020. Recruitment occurred in random time samples. During the random time samples, all eligible patients were asked to participate. Patients were included if they had been diagnosed with any type of cancer, were aged 18 years, and had sufficient oral and written proficiency in German language. Participants were informed that their medical data would be included in the evaluation of the study. Medical data were routinely collected on the first day and last day of the clinic stay. Afterward, the medical director distributed the pen-and-paper questionnaire to the participants, which the participants completed and handed to their treating physician the next day.

### Measures and Data Source

#### Cross-sectional Questionnaire

##### Sociodemographic and Medical Characteristics

The questionnaire during the last week of the clinic stay included multiple choice items designed to describe the sociodemographic (age, gender, years of schooling, professional situation, and current living situation) and medical characteristics (type of cancer) of the participants.

##### The Extent and Purpose of Patients’ Internet Use

We used an adapted version of the questionnaire used by Drewes et al [63] to measure the internet use of patients during their clinic stay and at home as well as their interest in future interactions with new media. First, the participants reported whether they used the internet. Participants who indicated not using the internet were instructed to skip all questions about the extent and purpose of internet use.

The frequency of internet use at home and during the clinic stay was self-reported by responses on a 4-point response scale from “never” to “daily.” Two items about the daily time spent on the web during the clinic stay and at home were answered on a 5-point response scale from “none” to “more than 120 minutes.” Furthermore, participants were asked which device they used to access the internet at home and during their clinic stay. To indicate the most common web-based activities during the clinic stay and at home, participants could select one or more of the 10 options of predefined activities and could enter an activity themselves.

##### Preferences for Future Use of eHealth or Web-Based Programs

Participants’ interests in future interactions with new media or web-based services in health care were determined by rating 6 statements on a 4-point Likert scale from “I strongly disagree” to “I strongly agree.”

##### Patients’ Views on Internet Use During Clinic Stay

Participants rated the following statements on a 4-point Likert scale from “I strongly disagree” to “I strongly agree”: “The availability of Wireless LAN (WLAN) in the rehabilitation clinic is very important to me,” “I would like to receive online support during treatment,” “I feel distracted from rehabilitation by using the internet during rehabilitation,” “I can fulfill my information needs by using the internet during my rehabilitation stay,” and “I was absent from the clinic’s leisure-time activities because I spent the time on the internet.”

##### Perceived Social Support Between Patients During Clinic Stay

To measure the perceived social support between patients during the clinic stay, the questionnaire on social support between patients (F-SozU-P) was used [64]. The F-SozU-P is an adaptation of the German self-report questionnaire for the assessment of social support (F-SozU) [65], which is the long version of the brief form for assessing social support (F-SozU K-6) [64]. Both the order and the sentence structure of the F-SozU items were retained in the F-SozU-P. However, words such as “people,” “relatives,” and “family” in the F-SozU were replaced by “fellow patients” or “patients” in the F-SozU-P. All 54 items were scored on a 5-point Likert scale ranging from 1=“not true” to 5=“exactly true.” In the validation study, the global scale wahrgenommene soziale Unterstutzung–Patienten (perceived social support-patients; WasU-P) had high values for internal consistency (α=.93) [64].

##### Physical Activity During Clinic Stay

Physical activity during the clinic stay was measured using the German version of the Godin-Shephard Leisure-Time Physical Activity Questionnaire (GSLTPAQ) [66]. The GSLTPAQ is commonly used for classification purposes in oncology [67]. Participants reported how often and how long (in minutes) they engaged in low-, moderate-, and high-intensity physical activity in the past week. The frequency at each intensity was multiplied by 3, 5, and 9 metabolic equivalents and then multiplied by the duration divided by 60 and summed. Scores derived from the GSLTPAQ represent the time of physical activity during the clinic stay in the form of metabolic equivalents hours within the last week [67].

#### Longitudinal Questionnaire

The longitudinal questionnaire included 3 validated PROMs. First, the German version of the Distress Thermometer [68] consists of a single-item scale ranging from 0=no distress to 10=extreme distress, indicating how much stress the participant experienced in the last week, including the day of assessment. A score of 5 is internationally recommended as an indicator that a patient is distressed and may need support [68]. Second, the German version of the numeric rating scale (NRS) for pain [69] is an 11-point numeric scale (NRS 11) ranging from 0=no pain to 10=worst pain imaginable [69]. This instrument is commonly used to measure pain in patients with cancer [7]. Third, participants completed the German version of the Brief Fatigue Inventory [70]. The Brief Fatigue Inventory is used for the specific assessment of fatigue in patients with oncological diseases. The questionnaire contains 10 items. Three items ask patients to rate the severity of their fatigue on average, at its worst, and right now, with 0=no fatigue and 10=fatigue as bad as you can imagine. In addition, 6 items measure the extent to which patients’ fatigue interferes with general activity, mood, walking, work, relationships with others, and enjoyment of life. These items are rated on a scale of 0=does not interfere to 10=completely interferes [70]. A score between 3 and 4 points indicates medium-severity fatigue in patients with tumors.

### Pilot Testing

We pilot-tested the complete set of items in March 2018 in 6 patients undergoing rehabilitation. The pilot participants were recruited from the same German rehabilitation clinic as the respondents in the following study. The inclusion criteria for participation in the pilot test were identical to those of the main study. Participants were instructed to think aloud while completing the questionnaires to identify how they interpreted items, whether instructions were easy to understand, whether problems occurred, and whether they understood the items in the way they were intended [71]. The pilot study showed satisfactory results and revealed that participants generally understood the set of items well. The completion of the questionnaire took between 25 and 50 minutes.

### Data Analysis

We used SPSS Statistics (version 25; IBM SPSS Inc) for the statistical analyses. The participants’ sociodemographic and medical characteristics, the extent and purpose of rehabilitant internet use, and their preferences for future use of eHealth or web-based programs were summarized descriptively (ie, means, SDs, frequencies, and percentages).

For further analysis, we excluded cases with >30% of missing F-SozU-P items [72]. We used multiple linear regression analysis to determine the association between the extent of participants’ internet use (independent variable) and perceived social support among patients during their clinic stay (dependent variable; research question 1). To identify whether participants who used the internet for interactive activities, such as “communication with relatives” and “writing emails,” reported more social support among them than patients who did not, a dummy-coded variable was included as an independent variable. To control for potential confounding variables, we included physical activity during the clinical stay (GSLTPAQ score), age, education (>10 years of school education vs ≤10 years), and sex as additional independent variables. Categorical variables were dummy coded. The variable extent of internet use was the product of 2 factors: the time spent on the web and the frequency of internet use during the clinic stay. To identify the extent of multicollinearity, the variance inflation factor (VIF) of all independent variables were reported. If the VIF is >10, there is reason for concern [73]. Missing values of the F-SozU-P and the independent variables, namely, the extent of internet use during rehabilitation, GSLTPAQ, age, and education were imputed using the expectation-maximization algorithm [74].

We used 3 linear mixed models with random intercepts to determine the association between the extent of participants’ internet use during inpatient rehabilitation (independent variable) and the change in distress as the primary outcome as well as the secondary outcomes, namely, fatigue and pain (dependent variables) from the beginning to the end of inpatient rehabilitation (research question 2). The dependent variables in each model were calculated as the difference between the outcomes on the first day and the last day of the clinic stay. To answer the research question, we tested the main effects of the extent of internet use (fixed factor). Furthermore, we included the fixed factors of social support among patients and the interaction between internet use and social support to test whether social support moderated the association between the extent of participants’ internet use and changes in the 3 PROMs. The variables of social support among patients and internet use were mean centered to avoid multicollinearity problems [75,76]. To identify the extent of multicollinearity, the VIFs of all fixed factors were reported using the R package “performance” (version 0.10.2; R Foundation for Statistical Computing) [77]. If the VIF is >10, there is reason for concern [73]. To control for differences in the baseline values and regression to the mean, baseline PROMs values were included as fixed factors [78,79]. The overall fit of the models was evaluated by the −2 log likelihood. We used the restricted maximum likelihood method to estimate the parameters in all 3 models [80].

Furthermore, we conducted a sensitivity analysis using the described 3 linear models before including the interaction term.

For the planned multiple regression analyses, we conducted an a priori power calculation by using G*Power [81]. On the basis of this analysis, we concluded that study data from 352 patients would be needed to sufficiently demonstrate a correlation with a small to medium effect size of *R*=0.20 (corresponding to an f-square=0.0417), with 80% power and a level of significance set at α=.05 in a multiple linear regression analysis with 7 predictor variables.

### Ethics Approval

The study was conducted in accordance with the Code of Ethics of the Declaration of Helsinki and was surveyed by the Ethics Committee of the local Medical Association (Schleswig-Holstein, Germany; study ID 042/18 II). Participants had to sign an informed consent form before they could participate in the study. The form included information about the study goal, potential risks and benefits of study participation, the voluntary nature of participation, and the type and duration of data storage.

## Results

### Cross-sectional Results

#### Participants Sociodemographic and Medical Characteristics

A total of 900 patients undergoing rehabilitation participated in this study; of them, 323 patients were asked to participate, which resulted in a response rate of 35.9% (323/900). The participants’ ages ranged from 29 to 88 years (Table 1). More female (172/323, 53.3%) than male patients participated in the study. Approximately one-third of the participants (111/323, 34.4%) had >10 years of school education. Almost half of the participants (146/323, 48.3%) were retired. Furthermore, 69.9% (226/323) of the participants were married or lived in a committed relationship. Colon (69/323, 17.5%), breast (66/323, 16.7%), and prostate (49/323, 12.4%) cancers were the most common types of cancer among the participants.

**Table 1:** Medical and sociodemographic characteristics of participants (N=323).

Participant characteristics		Values
Age (years), mean (SD, range)		62.3 (11.1, 29-88)
**Sex, n (%)**		
	Female	172 (53.3)
	Male	150 (46.4)
	Missing values	1 (0.3)
**Highest educational achievement, n (%)**		
	13 years of school education	111 (34.4)
	10 years of school education	110 (34.1)
	9 years of school education	93 (28.8)
	No degree	2 (0.6)
	Other	3 (0.9)
	Missing values	4 (1.2)
**Professional situation, n (%)^a^**		
	Retired	144 (44.6)
	Working full time	97 (30)
	Working part time	48 (14.7)
	Unemployed	11 (3.4)
	Housewife or househusband	18 (5.6)
	Other	20 (6.2)
	Missing values	0 (0)
**Current living situation, n (%)**		
	Living with partner or living with partner and children	222 (68.7)
	Living alone	83 (25.7)
	Living alone with kids	11 (3.4)
	Other	3 (0.9)
	Missing values	4 (1.2)
**Cancer type, n (%)^a^**		
	Colon	68 (21.1)
	Breast	65 (20.1)
	Prostate	49 (15.2)
	Lung	27 (8.4)
	Non-Hodgkin	24 (7.4)
	Kidney	18 (5.6)
	Mouth, throat, and esophagus	15 (4.6)
	Pancreatic	14 (4.3)
	Other	83 (25.7)
	Missing values	0 (0)

^a^Multiple selection; percentages of respondents.

#### The Extent and Purpose of Patients’ Use of the Internet During Their Clinic Stay and at Home

Of the 323 participants, 279 (86.4%) reported using the internet. These participants are referred to as “internet users” in the following section. During their clinical stay, 70.9% (198/279) of the internet users used the internet daily. At home, 84.9% (237/279) of the internet users used the internet daily (Table 2). Overall, 30 of the 279 (10.8%) internet users never used the internet during their clinic stay. During their clinic stay, 27 of the 279 (9.8%) internet users used the internet for more than 1 hour per day, compared with 84 of the 277 (30.3%) participants at home. Smartphones were the most frequently used device for internet access during the clinic stay (219/279, 78.4%) and at home (215/279, 77.1%). During the clinic stay and at home, social media use (192/279, 68.9%; 208/279, 74.6%) and emailing (143/279, 51.3%; 228/279, 81.7%) were among the 3 most frequently reported web-based activities.

**Table 2:** The extent and purpose of patients’ use of the internet during their clinical stay and at home (N=279).

Participant characteristics		Setting, n (%)	
		During clinic stay	At home
**Frequency of internet use**			
	Daily	198 (71)	237 (84.9)
	>Once a week	16 (5.7)	22 (7.9)
	>Once a month	0 (0)	5 (1.8)
	Rarely	23 (8.2)	11 (3.9)
	Never	30 (10.8)	2 (0.7)
	Missing values	12 (4.3)	2 (0.7)
**Daily time spent on the web in minutes**			
	>120	7 (2.5)	28 (10)
	60-120	20 (7.2)	56 (20)
	30-60	84 (30.1)	118 (36.5)
	0-30	119 (44.4)	71 (42.3)
	None	38 (13.6)	3 (1.1)
	Missing values	11 (3.9)	3 (1.1)
**Devices used to access the internet^a^**			
	Smartphone	219 (78.5)	215 (77.1)
	Tablet	69 (24.7)	118 (42.2)
	Laptop	62 (22.2)	152 (54.5)
	PC	4 (1.4)	130 (46.6)
	None	26 (9.3)	4 (1.4)
	Other	7 (1.4)	5 (1.8)
	Missing values	4 (1.4)	1 (0.3)
**Web-based activities^a^**			
	Using social media	192 (68.9)	208 (74.6)
	Communication with relatives	148 (53)	154 (55.2)
	Writing emails	143 (51.3)	228 (81.7)
	Other (news, web-based games, shopping on eBay or Amazon, erotic, etc)	106 (38)	205 (73.5)
	Searching for health-related information	68 (24)	173 (62)
	Reading	54 (19.4)	92 (33)
	Working	14 (5.0)	74 (26.5)
	Learning or studying	12 (4.3)	88 (31.5)
	Looking for treatment support	11 (3.9)	22 (7.9)
	Participation in web-based courses for private education and qualification	3 (1.1)	22 (7.9)
	Other	12 (4.3)	25 (9)
	Missing values	7 (2.5)	2 (0.7)

^a^Multiple selection; percentages of respondents.

#### Internet Users’ Views on Internet Use During the Clinic Stay and Patients’ Interest in Future Interaction With New Media or Web-Based Service in Health Care

About 9.3% (26/279) of internet users did feel distracted from rehabilitation by using the internet during their clinical stay, and 1.8% (5/279) reported having missed their clinic’s leisure-time activities because they spent time on the internet (Multimedia Appendix 2). The results concerning patients’ interest in future interactions with new media or web-based services in health care are displayed in Multimedia Appendix 3.

#### Association Between the Extent of Internet Use and Social Support Among Rehabilitants During Rehabilitation

A total of 2.2% (7/323) of cases were excluded from the multiple regression analysis because >30% of F-SozU-P items were missing. The mean perceived social support between patients during their clinic stay was 3.2 (SD 0.7).

The extent of internet use (*t*_315_=0.78; *P*=.43) was not significantly negatively associated with the perceived social support among the participants during their clinic stays (Table 3). Participants who were younger (*t*_315_=−6.01; *P*<.001) and female participants (*t*_315_=2.02; *P*=.04) perceived significantly more social support from other patients with cancer during their clinic stay than older and male participants, controlling for all other predictors in the model. Seventeen percent (*R*^2^=.17) of the variance in perceived social support among patients during rehabilitation was explained by the model. Participants who used the internet for communicative activities did not perceive more social support from other patients with cancer during their clinic stay (*t*_315_=−0.03; *P*=.98) than the participants who did not use it for communicative activities. The VIFs of the predictors ranged from 1.04 to 1.41.

**Table 3:** Parameters of the multiple regression analysis with perceived social support as the dependent variable (n=316).

Variables	b (SE)	2-tailed *t* test	*P* value	95% CI	VIF^a^
Intercept	4.31 (0.30)	14.17	<.001	3.71 to 4.90	—^b^
Extent of internet use during clinic stay	0.01 (0.01)	0.72	.43	−0.01 to 0.03	1.73
Age	−0.02 (0.00)	−5.95	<.001	−0.03 to −0.02	1.46
Sex (male vs female)	0.14 (0.07)	2.00	.04	0.00 to 0.28	1.09
GSLTPAQ^c^	0.00 (0.00)	0.81	.42	−0.00 to 0.01	1.05
Education (>10 years vs ≤10 years of school education)	−0.04 (0.07)	−0.57	.58	−0.18 to 0.10	1.05
Interactive internet use (users vs nonusers)	−0.00 (0.08)	−0.03	.98	−0.16 to 0.16	1.41

^a^VIF: variance inflation factor.

^b^Not available.

^c^GSLTPAQ: Godin-Shephard Leisure-Time Physical Activity Questionnaire.

### Longitudinal Results

#### Descriptive Overview for Both Measurement Points

Participants’ mean level of distress decreased from 5.2 (SD 2.4) to 2.7 (SD 2.1) from the beginning to the end of rehabilitation (Table 4). The mean fatigue decreased from 3.2 (SD 1.9) to 2.1 (SD 1.6) from the beginning to the end of rehabilitation. The mean pain decreased from 2.4 (SD 2.8) to 1.2 (SD 1.9) from the beginning to the end of rehabilitation.

**Table 4:** Descriptive data for outcomes for both measurement points (N=323).

Questionnaire	First measurement point			Second measurement point	
	Value, n (%)	Value, mean (SD)	Value, n (%)		Value, mean (SD)
DT^a^	315 (97.5)	5.2 (2.4)	311 (96.3)		2.7 (2.1)
BFI^b^	315 (97.5)	3.2 (1.9)	311 (96.3)		2.1 (1.6)
NRS^c^ for pain	316 (97.8)	2.4 (2.8)	315 (97.5)		1.2 (1.9)

^a^DT: Distress Thermometer.

^b^BFI: Brief Fatigue Inventory.

^c^NRS: numeric rating scale.

#### Association Between the Extent of Internet Use and Changes in Distress From the First to the Last Day of the Clinic Stay (Primary Outcome)

The extent of participants’ internet use during their clinic stay (*F*_1,299_=0.12; *P*=.73) and the perceived social support among patients (*F*_1,299_=2.69; *P*=.10) were not significantly associated with changes in participants’ distress levels (Multimedia Appendix 4). The interaction between the extent of participants’ internet use during their clinic stay and perceived social support among patients (*F*_1,299_=0.31; *P*=.58) was not significantly associated with changes in the participants’ distress levels. Higher baseline distress levels were significantly (*F*_1,299_=168.87; *P*≤.001) associated with greater changes in the participants’ distress levels. The VIFs of the fixed factors ranged from 1.01 to 1.07.

#### Association Between the Extent of Internet Use and Changes in Fatigue and in Pain From the First to the Last Day of the Clinic Stay (Secondary Outcomes)

The extent of participants’ internet use during their clinic stay (*F*_1,299_=0.19; *P*=.67) and the perceived social support among patients (*F*_1,299_=1.68; *P*=.20) were not significantly associated with changes in participants’ fatigue levels (Multimedia Appendix 5). The interaction between the extent of participants’ internet use during their clinic stay and perceived social support among patients (*F*_1,299_=0.12; *P*=.73) was not significantly associated with changes in the participants’ fatigue levels. Higher baseline fatigue levels were significantly (*F*_1,299_=143.10; *P*<.001) associated with greater changes in the participants’ fatigue levels. The VIFs of the fixed factors ranged from 1.01 to 1.07.

The extent of participants’ internet use during their clinic stay (*F*_1,303_=0.92; *P*=.34) and the perceived social support among participants (*F*_1,303_=0.35; *P*=.55) were not significantly negatively associated with changes in their pain levels (Multimedia Appendix 6). The interaction between the extent of participants’ internet use during their clinic stay and perceived social support among patients (*F*_1,303_=0.52; *P*=.47) was not significantly associated with changes in the participants’ pain levels. Higher baseline pain levels were significantly (*F*_1,303_=363.76; *P*≤.001) associated with greater changes in the participants’ pain levels. The VIFs of the fixed factors ranged from 1.01 to 1.07.

#### Sensitivity Analyses

Multimedia Appendix 7 summarizes the results of the 3 linear mixed models before including the interaction effects. The main effects for social support between patients and the extent of internet use did not change when the interaction term between the 2 variables was included.

## Discussion

### Principal Findings

The study results do not support the observations of health care professionals. The extent of internet use was not negatively associated with the perceived social support among patients with cancer during their stay at the oncological rehabilitation clinic. In addition, the extent of participants’ internet use during their clinic stay was not negatively associated with the change in the 3 PROMs, namely, distress (primary outcome), pain, and fatigue from the first day to the last day of the clinical stay. The results of this study represent the first examination of the associations between the extent of internet use, social support, and changes in rehabilitation outcomes in an inpatient rehabilitation setting.

Furthermore, the results of the multiple linear regression analysis indicate that younger and female participants perceived significantly more social support from other patients with cancer during their clinic stay than older and male participants.

The descriptive study results indicate that more than four-fifths of the patients with cancer were internet users. During clinic stay, 70.9% (198/279) of internet users used the internet daily. 10.8% (30/279) of the internet users never used the internet during their clinic stay.

### Comparison With Previous Work

The assumption before the start of the study was that a high level of internet use during rehabilitation could reduce social interaction between patients and, therefore, the perceived social support among patients with cancer during their clinic stay. This assumption was based on the observations of health care professionals and related to the social displacement hypothesis [50,52]. However, finding no association between the extent of participants’ internet use and perceived social support is consistent with the results of cross-sectional and longitudinal studies that examined internet use in healthy individuals [52-54] and in patients with spinal cord injuries [82]. Furthermore, only 1.8% (5/279) of internet users reported missing clinic leisure-time activities because they spent time on the internet. Finding female sex to be associated with more perceived social support from other patients in the clinic fits the results of the validation study of the F-SozU-P, in which female psychosomatic patients in inpatient rehabilitation perceived more social support than male patients [64]. Women seem to provide more emotional support to both men and women, and they seem to receive more help in return [24]. A positive association between younger age and higher perceived social support for patients with cancer may be partially explained by the findings of a previous study that reported that older adults reported seeking less explicit social support but reported using a similar amount of implicit social support, seeking to cope with their stressors [21]. In an unfamiliar environment with initially unfamiliar fellow patients, explicitly asking for emotional support seems to be associated with higher perceived social support.

The finding of no association between the extent of participants’ internet use and the change in participants’ levels of distress, pain, and fatigue from the first day to the last day of their clinic stay is inconsistent with the health care professionals’ observations and assumptions but is consistent with participants’ perceptions of the relationship between internet use and rehabilitation activities and partially consistent with previous study results [55,58-60]. Health care professionals observed that high levels of internet use interfered with the patients’ rehabilitation program and competed with social interaction between patients during their clinic stay. However, only 9.3% (26/279) and 1.8% (5/279) of internet users, respectively, reported that they felt distracted from the rehabilitation program and that they missed recreational activities at the clinic because they spent time on the internet. Previous study results indicated that the overall extent of internet or social media use is not, or only marginally, associated with well-being [55] or changes in well-being [59], which is consistent with the results of our study. However, previous studies also indicated that the association between internet or social media use and well-being depends on the type of internet or social media use [58-60]. Our study results indicate that the participants who used the internet for communicative activities did not perceive more social support from other patients with cancer during their clinic stay than the participants who did not use it for communicative activities. However, we did not measure the extent of different types of internet activities. Measuring the extent of different types of internet activities might have led to positive associations, for example, between interactive internet or social media use and friends and family, social support, and well-being [52,53,58,59]. Further studies should be conducted to investigate the causal direction of these associations. These studies should also include personality and social skills of the participants [52].

Finding no association between social support and the change in participants’ levels of distress from the first day to the last day of their clinic stay is inconsistent with the results of systematic reviews examining peer support interventions for patients with cancer [27] and breast cancer [26]. The results of systematic reviews show that peer support interventions increase perceived distress, quality of life, emotional well-being, and psychosocial functioning of patients with cancer [26,27,83]. We have 2 possible explanations for the lack of association between social support and changes in participants’ distress. First, social support during the clinic stay predominantly occurs between treatment sessions, at meals, and during leisure-time activities. This type of social interaction is unmoderated and unstructured, which could have no or even adverse effects on quality of life and distress [26,30]. In the absence of moderation, or group structure, expressions of anger and fear, as well as discussions about death and dying can increase [30,31]. Second, emotional support is highly desired by patients with cancer and has positive influence on the patients’ well-being. It may be that emotional needs are best met by close friends and relatives of patients with cancer rather than by relative strangers in peer groups [83,84].

Health care professionals’ observations and assumptions and the social displacement hypothesis share the implicit mediation hypothesis that social support mediates the effect of the extent of internet use on change in well-being. Because we found no association between the extent of internet use and the mediator social support in the multiple regression analysis, we assumed that the probability of finding a mediation was too low and therefore decided not to apply the mediation analysis [85]. In addition, social support did not moderate the association between the extent of participants’ internet use and changes in the 3 PROMs. Further studies should be conducted to examine the causal direction of these associations outside residential treatment.

This study is the first to present data on the extent and purpose of patients’ internet use during inpatient cancer rehabilitation. The prevalence of internet use among participants (279/323, 86.4%) was higher than that in previous studies with patients with cancer (60.2%-79.8%) [34,37,38] and very similar to the prevalence (87%) in the population of advanced economies [40]. The higher prevalence compared with previous studies with patients with cancer may be explained by the samples in the earlier studies being recruited in 2005 [34], 2007 [37], and 2015 [38] and the increasing internet access and use among patients with cancer [86].

### Limitations

The first limitation concerns the somewhat low participation rate, which could be an indicator that our sample had a nonresponse bias [87]. However, the scores of the study participants who experienced fatigue differed only slightly from the scores of all patients with cancer (n=1204) treated at the analyzed oncological rehabilitation clinic in 2019, indicating that our sample might be representative of patients in the rehabilitation clinic. Second, we were unable to find comprehensively validated instruments to measure perceived social support between patients, the extent and purpose of rehabilitating patients’ use of the internet, and patients’ interest in future interactions with web-based services. The F-SozU-P was validated as part of a dissertation project and showed good values for internal consistency and convergent and discriminant validity [64]. The items that we used to measure the extent and purpose of rehabilitating patients’ use of the internet and patients’ interest in future interactions with web-based services were obtained or adapted from a previous study by Drewes et al [63]. We pilot-tested all instruments of the questionnaire to assess the experiences of patients with cancer, while they were completing the instruments. The results of the pilot study showed that the participants generally understood the questions well, and no adjustments to the questionnaire had to be made. Third, 7.7% (25/323) to 12.1% (39/323) of the values for the items measuring patients’ interest in future interaction with web-based services were missing. The missing values can be partially explained by the fact that participants who reported not using the internet were instructed to skip all questions about the extent and purpose of internet use. Overall, of 44 noninternet users, 9 (20%) additionally skipped the last 6 questions of the questionnaire about their interest in future interactions with web-based services.

### Conclusions

The extent of internet use by patients with cancer during their clinic stay does not seem to be associated with the perceived social support among patients with cancer or with the change in their level of distress, fatigue, or pain from the first day to the last day of their clinic stay. Therefore, we recommend that clinics offer their patients free, easily accessible, and fast wireless local-area network connection.

## Appendix

Multimedia Appendix 1 The Strengthening the Reporting of Observational Studies in Epidemiology (STROBE) Statement.

Multimedia Appendix 2 Internet users' views on internet use during their clinic stay.

Multimedia Appendix 3 Participants’ interests in future interactions with new media or web-based services in health care.

Multimedia Appendix 4 Parameters of the linear mixed model analysis with distress as the dependent variable.

Multimedia Appendix 5 Parameters of the linear mixed model analysis with fatigue as the dependent variable.

Multimedia Appendix 6 Parameters of the linear mixed model analysis with pain as the dependent variable.

Multimedia Appendix 7 Parameters of the sensitivity analyses.

## Abbreviations

F-SozU-P: questionnaire on social support between patients
GSLTPAQ: Godin-Shephard Leisure-Time Physical Activity Questionnaire
NRS: numeric rating scale
PROM: patient-reported outcome measure
STROBE: Strengthening the Reporting of Observational Studies in Epidemiology
VIF: variance inflation factor
WasU-P: wahrgenommene soziale Unterstutzung–Patienten (perceived social support-patients)

## Data Availability

Our data are available on the Open Science Framework [88].
