# Supplementary material for: Association of the Extent of Internet Use by Patients With Cancer With Social Support Among Patients and Change in Patient-Reported Treatment Outcomes During Inpatient Rehabilitation: Cross-sectional and Longitudinal Study
Source: JMIR Cancer. 2023 May 17;9:e39246. doi: 10.2196/39246 (PMC10233445; doi:10.2196/39246)
Supplement: Multimedia Appendix 1 [file cancer_v9i1e39246_app1.docx]

STROBE Statement—checklist of items that should be included in reports of observational studies

|  | | Item No | Recommendation | Heading, Page  No |
| --- | --- | --- | --- | --- |
| **Title and abstract** | | 1 | (*a*) Indicate the study’s design with a commonly used term in the title or the abstract | Abstract, page 1 |
|  |  |  | (*b*) Provide in the abstract an informative and balanced summary of what was done and what was found | Abstract, pages 1-2 |
| Introduction | | | | |
| Background/rationale | | 2 | Explain the scientific background and rationale for the investigation being reported | Background, pages 2-5 |
| Objectives | | 3 | State specific objectives, including any prespecified hypotheses | Objectives, page 5 |
| Methods | | | | |
| Study design | | 4 | Present key elements of study design early in the paper | Study design, pages 6-7 |
| Setting | | 5 | Describe the setting, locations, and relevant dates, including periods of recruitment, exposure, follow-up, and data collection | Setting, Recruitment and Participants, page 7 |
| Participants | | 6 | *Cohort study*—Give the eligibility criteria, and the sources and methods of selection of participants. Describe methods of follow-up  *Case-control study*—Give the eligibility criteria, and the sources and methods of case ascertainment and control selection. Give the rationale for the choice of cases and controls  *Cross-sectional study*—Give the eligibility criteria, and the sources and methods of selection of participants | Setting, Recruitment and Participants, page 7 |
| Variables | | 7 | Clearly define all outcomes, exposures, predictors, potential confounders, and effect modifiers. Give diagnostic criteria, if applicable | Measures and data source, pages 7-10 |
| Data sources/ measurement | | 8* | For each variable of interest, give sources of data and details of methods of assessment (measurement). Describe comparability of assessment methods if there is more than one group | Measures and data source, pages 7-10 |
| Bias | | 9 | Describe any efforts to address potential sources of bias | Pilot Testing/Data Analysis, pages 10-12 |
| Study size | | 10 | Explain how the study size was arrived at | Data Analysis, page 11 |
| Quantitative variables | | 11 | Explain how quantitative variables were handled in the analyses. If applicable, describe which groupings were chosen and why | Data Analysis, 10-12 |
| Statistical methods | | 12 | (*a*) Describe all statistical methods, including those used to control for confounding | Data Analysis, 10-12 |
|  |  |  | (*b*) Describe any methods used to examine subgroups and interactions | Data Analysis, 10-12 |
|  |  |  | (*c*) Explain how missing data were addressed | Data Analysis, 10-12 |
|  |  |  | (*d*) *Cohort study*—If applicable, explain how loss to follow-up was addressed  *Case-control study*—If applicable, explain how matching of cases and controls was addressed  *Cross-sectional study*—If applicable, describe analytical methods taking account of sampling strategy | Data Analysis, 10-12 |
|  |  |  | (*e*) Describe any sensitivity analyses | Not applicable |
| Results | | | | |
| Participants | 13* | (a) Report numbers of individuals at each stage of study—eg numbers potentially eligible, examined for eligibility, confirmed eligible, included in the study, completing follow-up, and analysed | | Longitudinal Results, page 17 |
|  |  | (b) Give reasons for non-participation at each stage | | Not applicable |
|  |  | (c) Consider use of a flow diagram | | Not applicable |
| Descriptive data | 14* | (a) Give characteristics of study participants (eg demographic, clinical, social) and information on exposures and potential confounders | | Participants Sociodemographic and Medical Characteristics, page 12 |
|  |  | (b) Indicate number of participants with missing data for each variable of interest | | Tables 1-5 |
|  |  | (c) *Cohort study*—Summarise follow-up time (eg, average and total amount) | | Not applicable |
| Outcome data | 15* | *Cohort study*—Report numbers of outcome events or summary measures over time | | Not applicable |
|  |  | *Case-control study—*Report numbers in each exposure category, or summary measures of exposure | | Not applicable |
|  |  | *Cross-sectional study—*Report numbers of outcome events or summary measures | | Cross-Sectional Results, pages 12-17 |
| Main results | 16 | (*a*) Give unadjusted estimates and, if applicable, confounder-adjusted estimates and their precision (eg, 95% confidence interval). Make clear which confounders were adjusted for and why they were included | | Association between the Extent of Internet Use and Social Support Among Rehabilitants During Rehabilitation, page 19 |
|  |  | (*b*) Report category boundaries when continuous variables were categorized | | Not applicable |
|  |  | (*c*) If relevant, consider translating estimates of relative risk into absolute risk for a meaningful time period | | Not applicable |
| Other analyses | 17 | Report other analyses done—eg analyses of subgroups and interactions, and sensitivity analyses | | Association Between the Extent of Internet Use and Changes in Distress from the First to the Last Day of the Clinic Stay (Primary Outcome), pages 19-20 |
| Discussion | | | | |
| Key results | 18 | Summarise key results with reference to study objectives | | Principal Findings, page 20-21 |
| Limitations | 19 | Discuss limitations of the study, taking into account sources of potential bias or imprecision. Discuss both direction and magnitude of any potential bias | | Limitations, pages 23-24 |
| Interpretation | 20 | Give a cautious overall interpretation of results considering objectives, limitations, multiplicity of analyses, results from similar studies, and other relevant evidence | | Comparison with Previous Work, pages 20-22 |
| Generalisability | 21 | Discuss the generalisability (external validity) of the study results | | Limitations, page 21-23 |
| Other information | | | | |
| Funding | 22 | Give the source of funding and the role of the funders for the present study and, if applicable, for the original study on which the present article is based | | Acknowledgments, page 24 |

*Give information separately for cases and controls in case-control studies and, if applicable, for exposed and unexposed groups in cohort and cross-sectional studies.

**Note:** An Explanation and Elaboration article discusses each checklist item and gives methodological background and published examples of transparent reporting. The STROBE checklist is best used in conjunction with this article (freely available on the Web sites of PLoS Medicine at http://www.plosmedicine.org/, Annals of Internal Medicine at http://www.annals.org/, and Epidemiology at http://www.epidem.com/). Information on the STROBE Initiative is available at www.strobe-statement.org.
