# Supplementary material for: Association of the Extent of Internet Use by Patients With Cancer With Social Support Among Patients and Change in Patient-Reported Treatment Outcomes During Inpatient Rehabilitation: Cross-sectional and Longitudinal Study
Source: JMIR Cancer. 2023 May 17;9:e39246. doi: 10.2196/39246 (PMC10233445; doi:10.2196/39246)
Supplement: Multimedia Appendix 2 [file cancer_v9i1e39246_app2.docx]

**Multimedia Appendix 2.** Internet users' views on internet use during clinic stay.

| The availability of WLAN in the clinic is very important to me, n (%) | | |
| --- | --- | --- |
|  | Totally Agree/ Agree | 219 (78.5) |
|  | Totally Disagree/ Disagree | 52 (18.6) |
|  | Missing Values | 8 (2.9) |
| I would like to receive online support during treatment, n (%) | | |
|  | Totally Agree/ Agree | 60 (21.5) |
|  | Totally Disagree/ Disagree | 192 (68.8) |
|  | Missing Values | 27 (9.7) |
| I feel distracted from rehabilitation by using the Internet during my clinic stay, n (%) | | |
|  | Totally Agree/ Agree | 26 (9.3) |
|  | Totally Disagree/ Disagree | 233 (83.5) |
|  | Missing Values | 20 (7.2) |
| I can fulfill my information needs by using the internet during my clinic stay, n (%) | | |
|  | Totally Agree/ Agree | 95 (34.1) |
|  | Totally Disagree/ Disagree | 162 (58.1) |
|  | Missing values | 22 (7.9) |
| I feel empowered in managing my illness by using the internet during my clinic stay, n (%) | | |
|  | Totally Agree/ Agree | 72 (25.8) |
|  | Totally Disagree/ Disagree | 182 (65.2) |
|  | Missing Values | 25 (9.0) |
| I was absent from the clinic's leisure time activities because I spent the time on the internet, n (%) | | |
|  | Totally Agree/ Agree | 5 (1.8) |
|  | Totally Disagree/ Disagree | 257 (92.1) |
|  | Missing Values | 17 (6.1) |
