# Supplementary material for: Association of the Extent of Internet Use by Patients With Cancer With Social Support Among Patients and Change in Patient-Reported Treatment Outcomes During Inpatient Rehabilitation: Cross-sectional and Longitudinal Study
Source: JMIR Cancer. 2023 May 17;9:e39246. doi: 10.2196/39246 (PMC10233445; doi:10.2196/39246)
Supplement: Multimedia Appendix 3 [file cancer_v9i1e39246_app3.docx]

**Multimedia Appendix 3**. Participants’ interests in future interaction with new media or web-based services in health care (N=323).

| Participants’ interests | | Values |
| --- | --- | --- |
| I wish to use a smartphone for my health care, n (%) | | |
|  | Totally Agree/ Agree | 116 (35.9) |
|  | Totally Disagree/ Disagree | 182 (56.4) |
|  | Missing values | 25 (7.7) |
| I would like to receive therapy support via smartphone, n (%) | | |
|  | Totally Agree/ Agree | 99 (30.7) |
|  | Totally Disagree/ Disagree | 189 (58.5) |
|  | Missing values | 35 (10.8) |
| I would like to receive therapy support via PC or laptop, n (%) | | |
|  | Totally Agree/ Agree | 93 (28.8) |
|  | Totally Disagree/ Disagree | 194 (60.0) |
|  | Missing values | 36 (11.2) |
| I would like to receive therapy support via telephone, n (%) | | |
|  | Totally Agree/ Agree | 36 (11.2) |
|  | Totally Disagree/ Disagree | 248 (76.8) |
|  | Missing values | 39 (12.0) |
| I would like to document symptoms and side effects of my disease and treatment online, n (%) | | |
|  | Totally Agree/ Agree | 77 (23.8) |
|  | Totally Disagree/ Disagree | 214 (66.3) |
|  | Missing values | 32 (9.9) |
| I would like to document symptoms and side effects of my disease and treatment in an app, n (%) | | |
|  | Totally Agree/ Agree | 59 (18.3) |
|  | Totally Disagree/ Disagree | 230 (71.2) |
|  | Missing values | 34 (10.5) |
