# Supplementary material for: Association of the Extent of Internet Use by Patients With Cancer With Social Support Among Patients and Change in Patient-Reported Treatment Outcomes During Inpatient Rehabilitation: Cross-sectional and Longitudinal Study
Source: JMIR Cancer. 2023 May 17;9:e39246. doi: 10.2196/39246 (PMC10233445; doi:10.2196/39246)
Supplement: Multimedia Appendix 4 [file cancer_v9i1e39246_app4.docx]

**Multimedia Appendix 4**. Parameters of the linear mixed model analysis with distress as the dependent variable.

| **Independent Variable** | **Estimate** | **SE** | **P-value** | **95% CI** | **VIF^a^** |
| --- | --- | --- | --- | --- | --- |
| Intercept | 0.55 | 0.26 | .03 | 0.04, 1.06 |  |
| Extent of internet use | -0.01 | 0.03 | .73 | -0.06, 0.05 | 1.07 |
| Social support among patients | -0.28 | 0.17 | .10 | -0.61, 0.05 | 1.05 |
| Extent of internet use * Social support among patients | -0.02 | 0.04 | .58 | -0.10, 0.06 | 1.03 |
| Distress level baseline | -0.58 | 0.04 | <.001 | -0.66, -0.49 | 1.01 |

^a^ variance inflation factors

-2 log- likelihood = 1262.25
