# Supplementary material for: Association of the Extent of Internet Use by Patients With Cancer With Social Support Among Patients and Change in Patient-Reported Treatment Outcomes During Inpatient Rehabilitation: Cross-sectional and Longitudinal Study
Source: JMIR Cancer. 2023 May 17;9:e39246. doi: 10.2196/39246 (PMC10233445; doi:10.2196/39246)
Supplement: Multimedia Appendix 5 [file cancer_v9i1e39246_app5.docx]

**Multimedia Appendix 5**. Parameters of the linear mixed model analysis with the BFI score as the dependent variable.

| **Independent Variable** | **Estimate** | **SE** | **P-value** | **95% CI** | **VIF^a^** |
| --- | --- | --- | --- | --- | --- |
| Intercept | 2.30 | 1.30 | .08 | -0.26, 4.86 |  |
| Extent of internet use (centered) | 0.07 | 0.18 | .43 | -0.27, 0.42 | 1.07 |
| Social support among patients | -1.36 | 1.05 | .20 | -3.42, 0.70 | 1.05 |
| Extent of internet use * Social support among patients | -0.08 | 0.25 | .73 | -0.58, 0.41 | 1.02 |
| Fatigue level baseline | -0.42 | 0.35 | <.001 | -0.48, - 0.35 | 1.01 |

^a^ variance inflation factors

-2 log- likelihood = 2355.43
