# Supplementary material for: Association of the Extent of Internet Use by Patients With Cancer With Social Support Among Patients and Change in Patient-Reported Treatment Outcomes During Inpatient Rehabilitation: Cross-sectional and Longitudinal Study
Source: JMIR Cancer. 2023 May 17;9:e39246. doi: 10.2196/39246 (PMC10233445; doi:10.2196/39246)
Supplement: Multimedia Appendix 6 [file cancer_v9i1e39246_app6.docx]

**Multimedia Appendix 6**. Parameters of the linear mixed model analysis with pain as the dependent variable.

| **Independent Variable** | **Estimate** | **SE** | **P-value** | **95% CI** | **VIF^a^** |
| --- | --- | --- | --- | --- | --- |
| Intercept | 0.01 | 0.09 | .94 | -0.17, 0.18 |  |
| Extent of internet use (centered) | 0.02 | 0.02 | .34 | -0.02, 0.05 | 1.07 |
| Social support among patients | -0.06 | 0.11 | .55 | -0.27, 0.15 | 1.05 |
| Extent of internet use * Social support among patients | -0.02 | 0.03 | .47 | -0.07, 0.03 | 1.03 |
| Pain level baseline | -0.48 | 0.03 | <.001 | -0.53, - 0.43 | 1.01 |

^a^ variance inflation factors

-2 log- likelihood = 1009.41
