# Supplementary material for: Association of the Extent of Internet Use by Patients With Cancer With Social Support Among Patients and Change in Patient-Reported Treatment Outcomes During Inpatient Rehabilitation: Cross-sectional and Longitudinal Study
Source: JMIR Cancer. 2023 May 17;9:e39246. doi: 10.2196/39246 (PMC10233445; doi:10.2196/39246)
Supplement: Multimedia Appendix 7 [file cancer_v9i1e39246_app7.docx]

**Multimedia Appendix 7**.

**Table 1.** Parameters of the linear mixed model analysis with distress as the dependent variable.

| **Independent Variable** | **Estimate** | **SE** | **P-value** | **95% CI** | **VIF^a^** |
| --- | --- | --- | --- | --- | --- |
| Intercept | 0.53 | 0.26 | .04 | 0.03, 1.04 |  |
| Extent of internet use | -0.01 | 0.03 | .67 | -0.07, 0.04 | 1.05 |
| Social support among patients | -0.27 | 0.17 | 0.11 | -0.61, 0.06 | 1.05 |
| Distress baseline | -0.58 | 0.04 | <.001 | -0.67, -0.49 | 1.00 |

^a^ variance inflation factors

-2 log- likelihood = 1257.99

**Table 2.** Parameters of the linear mixed model analysis with fatigue as the dependent variable.

| **Independent Variable** | **Estimate** | **SE** | **P-value** | **95% CI** | **VIF^a^** |
| --- | --- | --- | --- | --- | --- |
| Intercept | 2.25 | 1.29 | .08 | -0.29, 4.79 |  |
| Extent of internet use | 0.07 | 0.17 | .69 | -0.27, 0.41 | 1.05 |
| Social support among patients | -1.34 | 1.05 | 0.20 | -3.40, 0.71 | 1.05 |
| Fatigue baseline | -0.41 | 0.03 | <.001 | -0.48, -0.34 | 1.00 |

^a^ variance inflation factors

-2 log- likelihood = 2354.60

**Table 3.** Parameters of the linear mixed model analysis with pain as the dependent variable.

| **Independent Variable** | **Estimate** | **SE** | **P-value** | **95% CI** | **VIF^a^** |
| --- | --- | --- | --- | --- | --- |
| Intercept | 0.55 | 0.26 | .03 | 0.04, 1.06 |  |
| Extent of internet use | 0.02 | 0.02 | .38 | -0.02, 0.05 | 1.05 |
| Social support among patients | -0.06 | 0.11 | .57 | -0.27, 0.15 | 1.05 |
| Pain baseline | -0.48 | 0.03 | <.001 | -0.53, 0.43 | 1.00 |

^a^ variance inflation factors

-2 log- likelihood = 1262.25
